# Lower Bounds for Coulombic Systems

**DOI:** 10.1021/acs.jctc.0c01301

**Published:** 2021-02-26

**Authors:** Eli Pollak, Rocco Martinazzo

**Affiliations:** † Chemical and Biological Physics Department, Weizmann Institute of Science, 76100 Rehovot, Israel; ‡ Department of Chemistry, University of Milan and Institute of Molecular Science and Technologies (ISTM), Consiglio Nazionale delle Ricerche (CNR), I-20133 Milan, Italy

## Abstract

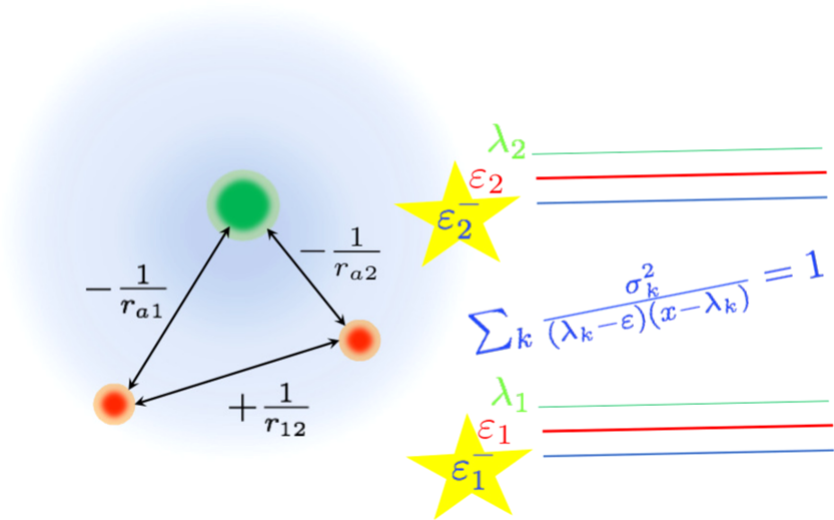

As of the writing of this paper, lower bounds are not a staple of quantum chemistry computations and for good reason. All previous attempts at applying lower bound theory to Coulombic systems led to lower bounds whose quality was inferior to the Ritz upper bounds so that their added value was minimal. Even our recent improvements upon Temple’s lower bound theory were limited to Lanczos basis sets and these are not available to atoms and molecules due to the Coulomb singularity. In the present paper, we overcome these problems by deriving a rather simple eigenvalue equation whose roots, under appropriate conditions, give lower bounds which are competitive with the Ritz upper bounds. The input for the theory is the Ritz eigenvalues and their variances; there is no need to compute the full matrix of the squared Hamiltonian. Along the way, we present a Cauchy–Schwartz inequality which underlies many aspects of lower bound theory. We also show that within the matrix Hamiltonian theory used here, the methods of Lehmann and our recent self-consistent lower bound theory (*J. Chem. Phys.***2020,***115,* 244110) are identical. Examples include implementation to the hydrogen and helium atoms.

## 1 Introduction

One of the outstanding challenges especially in ab initio quantum chemistry is obtaining lower bounds to atomic and molecular energies, which are as accurate as the upper bounds obtained with the Courant–Fischer theorem from the Ritz variational method.^1^ Lower bound methods abound, starting with Temple’s seminal expression derived in 1928.^2^ Landmarks in the derivation of lower bounds are Weinstein’s lower bound of 1934^3,4^ and Lehmann’s optimization of Temple’s lower bound presented in 1949–50.^5,6^ Especially Lehmann’s expression has turned out to be quite accurate in different settings, however not so for Coulombic systems, as exemplified by computations on the He^7−10^ and Li^11^ atoms.

In the past few years, we have presented an improvement of Temple’s formula for lower bounds of eigenvalues of Hermitian operators *Ĥ*(12−15) and like Lehmann’s theory it can become as accurate as the Ritz upper bound estimates.^16^ However, these results were derived through explicit use of a Lanczos basis set,^17^ which depends on a Krylov space,^18^ in which one creates basis vectors by repeated application of the Hamiltonian operator. This does not work for unscreened Coulomb potentials^19^ because the Coulomb singularity causes the third and higher moments of the Hamiltonian to diverge.

In this paper, we provide an answer to this challenge. Assuming an *L* dimensional subspace of the Hilbert space (as is standard in the Ritz variational theory), we present a lower bound expression, which depends only on the Ritz eigenvalues and associated variances. In contrast to Lehmann’s method, there is no need to compute the matrix representing *Ĥ*^2^ in the chosen space. The theory utilizes Lehmann’s approach as well as our recent results. To distinguish the present theory from previous ones, we will refer to it as the Pollak–Martinazzo (PM) lower bound theory using the abbreviation PM theory.

In Section 2, we review the known lower bound theories and their simplification when using Lanczos basis sets. Especially for Lehmann’s theory, we derive a new and simplified expression of the Lehmann eigenvalues, which replaces the necessity of knowing the full *Ĥ*^2^ matrix with the need to know only the variances of the Ritz eigenstates which, in turn, thanks to the Lanczos construct, are available from the Hamiltonian matrix only. We then construct in Section 3 an *L* + 1-dimensional auxiliary Hamiltonian matrix whose diagonal elements are the *L* Ritz eigenvalues. The additional dimension is taken to be such that the *L* + 1 dimensional matrix is parametrically dependent on one of its eigenvalues, which can be set at will, and in practice is set formally to one of the true (unknown) eigenvalues of the Hamiltonian operator. In this construct, the standard deviations associated with the Ritz eigenvalues turn out to be coupling elements, which couple the approximate eigenfunctions associated with the Ritz eigenvalues to the exact eigenfunction associated with the chosen exact eigenvalue. In Sections 3.2 and 3.3, we proceed to show that the resulting eigenvalue expression is consistent with both the Lehmann and our previous self-consistent lower bound theory when applied to the *L* + 1-dimensional auxiliary problem. The reader interested only in the new lower bound theory and its implementation can skip these at a first reading.

To apply the theory in practice, it is necessary to determine a lower bound to one of the roots of the eigenvalue expression. We show that this is readily obtained by considering how the roots of the equation change with increasing dimensionality. As a result, there is a parallel to the so-called Lehmann pole^20^ such that the Lehmann pole, which is employed in the “standard” Lehmann lower bound theory, turns out to be also the pole needed to construct lower bounds through the new lower bound expression. It cannot be over stressed that the “PM” theory derived in this paper depends only on the Ritz eigenvalues and their associated variances. Eigenfunctions associated with the Ritz eigenvalues are only needed for computation of the associated variances but there is no need to compute the full *Ĥ*^2^ matrix.

In Section 4, we apply the resulting theory to the He atom using a scaled Schrödinger basis set^25^ and to the hydrogen atom using a Gauss Hermite basis set. The resulting lower bounds are superior to estimates based on Temple’s expression and the “standard” Lehmann theory, which is not only less accurate but also considerably more expensive since it is based on computation of the *L* dimensional matrix of *Ĥ*^2^. We end with a discussion of the advantages, future challenges, and possible pitfalls in application of the new method to more complex atoms and molecules.

## 2 Short Review of Previous Lower Bound Theories

### 2.1 Framework

We start with a Hamiltonian operator, whose eigenvalues and eigenstates are denoted as
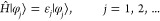
2.1

The eigenvalues are ordered in ascending order, that is, if *j* ≤ *k*, then ε_*j*_ ≤ ε_*k*_. The ground state is given the index 1 (rather than 0) to simplify the notation later on. We also assume the existence of a known orthonormal basis set |Ψ_*j*_⟩ = 1, 2, ... such that the Hamiltonian operator may be represented exactly as
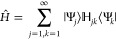
2.2and
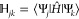
2.3

To simplify, we will assume that all functions and associated overlaps are real; however, this is not essential; the important property is that the operator under study is Hermitian. As in any practical computation, one never has the full Hamiltonian matrix (except for special cases) but rather its representation in a finite basis set, say the first *L* states spanning a space . Henceforth, in order to simplify notation, we will not indicate the dimensionality *L* and denote with  the projector onto  and with  that onto its orthogonal complement.

The Hamiltonian projected onto the finite basis set is
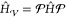
2.4and we assume that we know how to diagonalize this Hamiltonian in  such that it has eigenvalues λ_*j*_ and normalized eigenfunctions |Φ_*j*_⟩
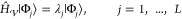
2.5

With each state, we also define a standard deviation σ_*j*_
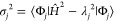
2.6

The overlap squared of the *j*th eigenfunction in the projected space with the exact *k*th eigenfunction is denoted as
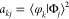
2.7

For future reference, we note that the variance may be rewritten as
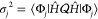
2.8

### 2.2 Weinstein and Temple Lower Bound Expressions

Underlying the derivation of many lower bounds is the following Cauchy–Schwartz inequality
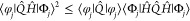
2.9where *Q̂* is a projector. For example, choosing
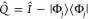
2.10inserting it into the Cauchy–Schwartz inequality and rearranging gives the inequality
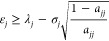
2.11

Assuming that *a*_*jj*_ ≥ 1/2 gives the Weinstein lower bound
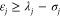
2.12

As discussed in ref (15), this assumption is somewhat less restrictive than the accepted condition for the validity of the Weinstein lower bound,^4^ which is that the Ritz eigenvalue λ_*j*_ is the closest one to the true eigenvalue ε_*j*_, that is,
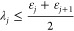
2.13

To derive the Temple lower bound, we define with each eigenstate in the projected space a “residual energy” λ̅_*j*_ such that
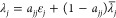
2.14

With this definition, the residual energy may be expressed in terms of the overlaps and exact eigenvalues as
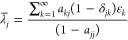
2.15where δ_*jk*_ is the Kronecker delta. It is a matter of straightforward algebra to show from eq 2.14 that
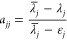
2.16

Inserting this identity into eq 2.11 and rearranging, one finds the Temple lower bound expression
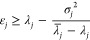
2.17

In the form of eq 2.17, the previous unknown overlap *a*_*jj*_ has been replaced by the as yet unknown residual energy λ̅_*j*_. However, we have gained something. Consider the ground-state residual energy. For any *k* ≥ 2, we have, through the initial ordering of the eigenvalues, the property ε_*k*_ ≥ ε_2_ so that
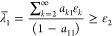
2.18

The Temple lower bound for the ground state now takes the well-known form
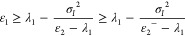
2.19where ε_2_^–^, which must be greater than λ_1_, is defined as a lower bound to the first excited-state energy. This lower bound may be obtained through a variety of lower bound methods such as the Weinstein,^3^ Bazley,^21,22^ Miller,^23^ and Marmorino^24^ methods. Introduction of the residual energy made it possible to obtain a practical calculable form of Temple’s lower bound formula.

If the Ritz eigenvalues converge to the exact energies when increasing the dimensionality of , so will the Weinstein and Temple lower bounds since the variance vanishes for exact eigenstates. However, the convergence will be much slower than the Ritz convergence due especially to the variances. As noted in ref (8), the integrand of a diagonal matrix element of the Hamiltonian squared is always positive so that all errors in the approximate wavefunction add up. This is not the case for the Hamiltonian itself, where positive and negative errors tend to cancel each other out, leading to more rapid convergence. It is this slow convergence of the Weinstein and Temple lower bounds, as exemplified by precise computations on the He atom,^8^ which has hindered their usage.

### 2.3 Self-Consistent Lower Bound Theory

Instead of using the crude separation of the Hilbert space as in eq 2.10, one may use the projector  onto the orthogonal complement to  and insert it into the Cauchy–Schwartz inequality (eq 2.9). Upon rearranging, this leads to an improved lower bound inequality
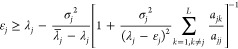
2.20

This result is superior to Temple’s lower bound since the expression in square brackets is always greater than unity. However, the overlaps *a*_*jk*_ are unknown. As shown in our previous papers,^14,15^ the key to turning this result into a practical one is the use of a Lanczos basis set so that the Hamiltonian has the form
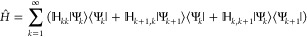
2.21

This implies that the “complementary part” of the Hamiltonian takes the form
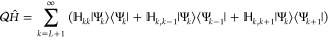
2.22so that, for example, from eq 2.8 one finds
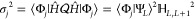
2.23

Using the identity
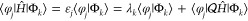
2.24one finds the needed relation
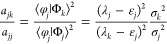
2.25

Inserting this into eq 2.20 gives a practical improved lower bound expression
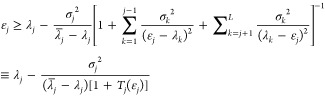
2.26

One also notes that the same considerations lead to an improved Weinstein lower bound. Assuming as before that *a*_*jj*_ ≥ 1/2 but using the projection operator  one readily finds that
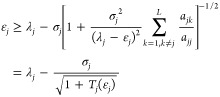
2.27where the second line is valid only when using a Lanczos basis set.

The implementation of these results and their improved convergence properties have been discussed in some detail in refs (14) and (15). The central drawback is that the expression is derived by using the Lanczos basis set, which does not exist for Coulombic systems due to the Coulomb singularity. It is this challenge which is addressed in this paper.

### 2.4 Lehmann Theory

The Temple lower bound as expressed in eq 2.17 is based on a particular choice of a basis function, namely, the eigenfunction of the Ritz eigenvalue. Lehmann noticed that one may choose a better linear combination of states in the space  by solving a generalized eigenproblem in this space. The Lehmann equation is, in a form that suits best our purposes,
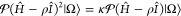
2.28where κ is the Lehmann eigenvalue and  is the associated Lehmann eigenfunction. The parameter ρ is known as the Lehmann pole and can be any real number but a Ritz eigenvalue for the above eigenproblem to be well-defined. However, for eq 2.28 to provide lower bounds τ = κ + ρ to the first L* ≤ *L* lowest eigenvalues (as is customarily needed in quantum chemistry calculations), the sample space  must be “good enough” such that λ_L*_ ≤ ε_L*+1_ holds, and ρ must be limited by the condition λ_L*_ ≤ ρ ≤ ε_L*+1_. Only under such circumstances will eq 2.28 deliver L* negative Lehmann eigenvalues and these are lower bounds to the first L* eigenvalues of *Ĥ*. In practice, then, L* is the highest state for which the inequality λ_L*_ ≤ ε_L*+1_ holds and ρ is a lower bound to ε_L*+1_.

To understand the lower bound property of the Lehmann bounds (the τ’s obtained from the Lehmann eigenvalues according to κ + ρ), it is useful to introduce
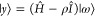
2.29for arbitrary  and to notice that the Lehmann equation amounts to the stationary condition of an ordinary Rayleigh–Ritz quotient involving the resolvent *G*(ρ) = (*Ĥ* – ρ*Î*)^−1^. Specifically, for |*y*⟩ arbitrary in the space , we have
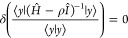
2.30if and only if |*y*⟩ ≡ |*Y*⟩ = (*Ĥ* – ρ*Î*)|Ω⟩ where |Ω⟩ satisfies eq 2.28, and in turn
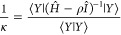
2.31where κ^–1^ is the corresponding quotient (a Ritz eigenvalue of *G*(ρ)). Then, the Courant–Fischer theorem guarantees that the negative values κ^–1^ are upper bounds to the exact eigenvalues (ε_*k*_ – ρ)^−1^ of *G*(ρ) from above for ε_*k*_ lower than ρ; if the negative κ’s are sorted in order of decreasing magnitude, |κ_L*_| ≤ |κ_L*–1_| ≤ |κ_1_|, then τ_*n*_ = ρ + κ_n_ is a lower bound to the (L* – *n* + 1)th eigenvalue left of ρ, that is the lower bounds are ordered as τ_*k*_ < ε_*k*_.

To see the connection with Temple’s lower bound, one multiplies eq 2.28 with the bra ⟨Ω| to find that
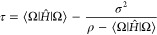
2.32with
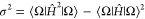
2.33

The Ritz variational theorem which underlies the Lehmann construct, as in eq 2.31, shows that the Lehmann eigenfunction is the function that maximizes Temple’s lower bound.

To summarize thus far, the Lehmann method builds on the matrices of *Ĥ*^2^ and *Ĥ* in the  space; diagonalization of eq 2.28 gives the lower bound eigenvalues. The condition that ρ ≤ ε_L*+1_ implies that one needs knowledge of a non-trivial lower bound to the state ε_L*+1_, this could be a Weinstein- or a Bazley-related lower bound.

Interestingly, when using a Lanczos basis, one does not need to know the full *Ĥ*^2^ matrix in the projected space but only the variances σ_*k*_^2^ associated with the respective Ritz eigenvalues. To see this, one multiplies eq 2.28 by the bra ⟨Φ_*k*_| to find
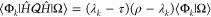
2.34so that
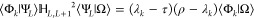
2.35

Multiplying by ⟨Ψ_*L*_|Φ_*k*_⟩ gives
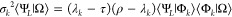
2.36

Rearranging and summing over all *k* from 1 to *L* gives an eigenvalue equation, valid for the Lanczos construct
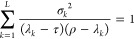
2.37and one notes expressly that the variances may be obtained from eq 2.23, that is, all the information is in matrix elements of the Hamiltonian only. The challenge then is to obtain similar results also in the case of Coulombic potentials where the Lanczos construct is not possible.

## 3 Lower Bounds for Coulombic Systems

### 3.1 Hamiltonian Matrix Construct for Lower Bounds

The “Achilles heel” in the simplifications presented in the previous section is the need to create a Lanczos basis with the full Hamiltonian. Of course, for a finite Hamiltonian matrix representation, any power of the matrix is well defined and does not diverge. As before, in the projected space , we assume that the *L*-dimensional Hamiltonian matrix is diagonal, with known Ritz eigenvalues and associated variances. At this point, we do not discuss how these variances are computed. We then expand the diagonal Hamiltonian matrix with one additional row and column such that it takes the form
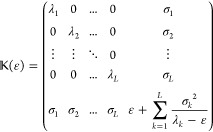
3.1where ε is for the time being a parameter. Notice that, for the sake of clarity, we use a simplified notation for ; henceforth, it is understood to have the dimension (*L* + 1) × (*L* + 1) where . In this “auxiliary” Hamiltonian matrix, the standard deviations σ_*j*_ couple the “Ritz states” to the added new state and, because of the Cauchy interlacing theorem,^26^ its eigenvalues *x*_*k*_ (*k* = 0, 1,..., *L*) are interlaced by the λ_*k*_’s, that is, *x*_*k*–1_ ≤ λ_*k*_ ≤ *x*_*k*_. Among these *L* + 1 new eigenvalues, one will be the energy ε. To see this, we note that the eigenvalue equation is
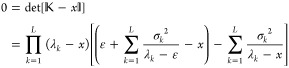
3.2

The expression in the square brackets has to vanish and this implies that
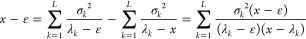
3.3and clearly one solution is *x* = ε. The other *L* eigenvalues^1^*x*_*j*_, *j* = 1, ..., *L* are the solutions of the remaining polynomial equation
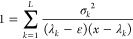
3.4

Notice the interesting symmetry: if *x*_*k*_ is an eigenvalue of  other than ε, then ε is an eigenvalue of  other than *x*_*k*_.

As we shall show below, eq 3.4 lies at the heart of PM theory. Lower estimates on the *x*_*k*_ poles will lead under suitable conditions to lower bounds to the eigenvalue under consideration. For example, when interested in the ground-state energy ε_1_, we find that one should expect that the first root *x*_1_ ≥ε_2_ so that a lower bound on the first excited-state energy ε_2_ will give a lower bound to the ground-state energy, provided, of course, that the Ritz eigenvalue for the ground state is lower than the lower bound for the first excited state. Note the formal similarity between eq 3.4 and the Lanczos-based Lehmann equation for the lower bound derived in eq 2.37. It is in this sense that PM theory generalizes Lehmann theory without the need to use a Lanczos basis set.

The eigenvalue eq 3.4 may also be rewritten as
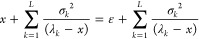
3.5from which one finds that
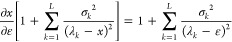
3.6or in other words the eigenvalues of  are increasing functions of the energy parameter ε.

Examination of eq 3.6 might suggest that this monotonicity is only in intervals since one encounters an infinity as either *x* or ε go through a Ritz eigenvalue. In reality, there is no discontinuity as ε comes close to a Ritz eigenvalue; the same will happen to all the roots x of eq 3.4 except one. The result is that the roots can be arranged to define functions *x*^(*k*)^(ε) that are continuous on the whole real axis except for a single pole singularity at a Ritz eigenvalue λ_*k*_ and which are monotonically increasing in each connected sub-domain (−∞, λ_*k*_), (λ_*k*_, +∞). This property is discussed further in detail in the Appendix where it is shown that the above-mentioned singularity is harmless for the method described below.

Monotonocity has far-reaching implications. Let us set ε = ε_1_ and consider the limiting situation where the Ritz eigenvalues λ_*k*_ approach the exact eigenenergies ε_*k*_ and have thus vanishingly small variances σ_*k*_^2^. We then choose the lowest *L* eigenvalues to construct the  space and the auxiliary matrix. In this limit, the matrix  has *L* eigenvalues matching the Ritz values and one eigenvalue which diverges to +∞ since the (*L* + 1)^th^ diagonal entry of the auxiliary matrix causes  to diverge. Having chosen one of the eigenvalues of  to be ε_1_, then, necessarily, all other roots of the eigenvalue eq 3.4 are such that *x*_*k*_(ε_1_) → λ_*k*+1_. Moreover, this occurs from below because of the interlacing theorem (λ_*k*_ ≤ *x*_*k*_(ε_1_) ≤ λ_*k*+1_). In addition, since with increasing accuracy each λ_*k*+1_ tends to ε_*k*+1_ from above the same holds for the roots *x*_*k*_(ε_1_), that is, ε_*k*+1_ ≤ *x*_*k*_(ε_1_) in this limit.

**Figure 1:**
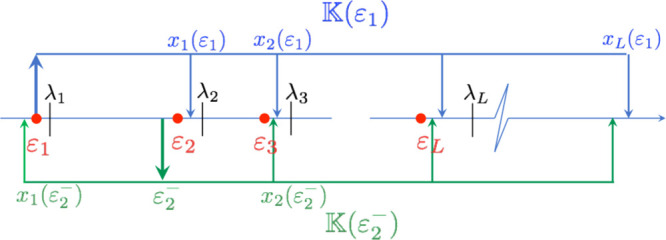
Diagram showing the use of the auxiliary matrix . The top part (blue) of the figure shows the spectrum of the matrix when the energy parameter ε = ε_1_ and the bottom (in green) when the energy parameter is chosen as a lower bound to the first excited-state energy (ε = ε_2_^–^). Red dots indicate the positions of the exact energy levels ε_*k*_ and black vertical bars that of the Ritz eigenvalues λ_*k*_. Note that *x*_1_(ε_2_^–^) is a lower bound to ε_1_ and *x*_1_(ε_1_) is an upper bound to ε_2_.

Now, we can revert the argument. Suppose that the calculation is sufficiently converged such that λ_*k*_ ≤ ε_*k*+1_ and that we know a rough lower bound (yet greater than λ_*k*_) to the (*k* + 1)th exact energy, call it ε_*k*+1_^–^. We know that there exists an ε such that  has ε_*k*+1_^–^ among its eigenvalues *x*_*k*_(ε). Indeed, thanks to the symmetry of eq 3.4, such an ε is just the lowest eigenvalue of  and—this is the key point—since *x*(ε) is monotonically increasing, ε is guaranteed to be left of (i.e., lower than) ε_1_. In other words, we have managed to convert a lower bound to the (*k* + 1)th energy into a lower bound to the ground-state energy. This is further shown in Figure 1 for the case *k* = 1. The question of whether this “map” produces a tight (hence useful) bound will be addressed numerically below, where it will be shown to be indeed the case.

The condition *x*_*k*_(ε_1_) > ε_*k*+1_ deserves some further comments since it is the key to obtaining lower bounds to the ground state. For definiteness, let us focus on the case *k* = 1 considered in the applications of Section 4. As shown in the Appendix, it is the degree of convergence of the ground-state Ritz eigenvalue that determines the closeness of *x*_1_ to λ_2_ irrespective of whether or not λ_2_ is close to ε_2_. Hence, if λ_1_ is reasonably close to ε_1_, then *x*_1_ should be close enough to λ_2_ to guarantee that *x*_1_ > ε_2_. As demonstrated for the computations of lower bounds for the He and H atoms, this property is verifiable by considering the dependence of *x*_1_ on the dimensionality *L* of the computation. If, and this is the typical case, it is a monotonically decreasing function of the dimensionality, then due to the limit that ultimately λ_2_ → ε_2_, the eigenvalue *x*_1_ is guaranteed to lie above ε_2_. The fact that ε_1_ is not known is not critical since the property of a monotonically decreasing value of *x*_1_ with increasing dimensionality will hold for a range of ε values close to ε_1_. It is this added property that distinguishes PM theory from Lehmann theory when the Lanczos construction is not exploited or not possible. When the latter is used, the ordering *x*_1_(ε_1_) > ε_2_ always holds and the two theories become equivalent to each other. In fact, if we had *x*_1_(ε_1_) < ε_2_, we could choose a pole ρ in eq 2.37 larger than *x*_1_ and yet below ε_2_ (*x*_1_ ≤ ρ ≤ ε_2_) and bound in this way the ground state from below. However, this is clearly impossible by virtue of monotonicity (eq 3.6) since ε_1_ < *y*_1_(ρ) where *y*_1_ is the inverse function of *x*_1_.

These same considerations can be generalized to excited states. For the sake of clarity, let us focus on the first excited state ε_2_ and set ε = ε_2_ in the auxiliary matrix. In the limiting situation considered above, the eigenvalues *x*_*k*_(ε_2_) approach the corresponding Ritz values from below, but now, due to the interleaving theorem and our ordering of the eigenvalues, *x*_1_(ε_2_) ≤ λ_1_ and *x*_*k*_(ε_2_) ≤ λ_*k*+1_ for 2 < *k* ≤ *L*. The lowest root—*x*_1_(ε_2_) is a lower bound to the ground state since, according to the above, we know that ε_2_ < *x*_1_(ε_1_) and we have to move *x*_1_ leftward to match ε_2_. The remaining eigenvalues, on the other hand, are upper bounds to the states higher than ε_2_ for the very same reasons given above. Hence, even a crude lower bound ε_*k*+1_^–^ can be converted into a lower bound to ε_2_^–^. As is usual when using a finite basis set, the quality of the Ritz eigenvalues deteriorates as one “goes up the eigenvalue ladder”. There will be some value L* above which the interleaving property of the Ritz and exact eigenvalues is no longer valid. However, the upper bound quality of the roots of the auxiliary Hamiltonian remains up to L*, that is, *x*_L*_ ≥ ε_L*_. In practice, then, one can use the highest index (*k* + 1 = L*) for which ε_L*_ ≥ λ_L*–1_ holds and use a lower bound ε_L*_^–^ (yet such that ε_L*_^–^ ≥ λ_L*–1_) to obtain lower bounds to all the lower lying states. This is the analogue of the pole in Lehmann theory.

The practical implementation of the PM theory parallels the practical implementation of the Lehmann theory. One starts with a valid lower bound to the Lehmann pole. Then, one computes all roots of eq 3.4 which are below the lower bound to the Lehmann pole and these will be lower bounds to the respective eigenvalues.

Apart from the increased lower bound accuracy obtained through the PM method, we note that the computational expense may be lower than the effort involved in computing the “standard” Lehmann lower bound. For the Lehmann equation, one needs the full *Ĥ*^2^ matrix. For the PM method, one only needs the variances associated with the Ritz eigenfunctions. This implies that if |Φ_*j*_⟩ is an eigenfunction, one can compute directly diagonal matrix elements of the sort ⟨Φ_*j*_|*Ĥ*^2^|Φ_*j*_⟩ and there is no need to first compute the *Ĥ*^2^ matrix.

In the next section, we will give a numerical example which shows that the present theory gives improved lower bounds for the excited states as compared to any other existing method.

### 3.2 Lehmann Eigenvalue Equation for the Hamiltonian of Equation 3.1

The result of the previous subsection has the flavor of the Lehmann lower bound and now we will show that indeed it is identical to it provided that one uses in the Lehmann eq 2.28 the matrix Hamiltonian  (eq 3.1) instead of the full Hamiltonian *Ĥ*. For this purpose, it is expedient to introduce an auxiliary vector |Ψ^⊥^⟩ (which is only required to be orthogonal to the |Φ_*k*_⟩ vectors, for *k* = 1, ..., *L*) and interpret the matrix  as the representation of an operator *K̂*(ε) acting in the enlarged, *L* + 1-dimensional space built with the |Φ_*k*_⟩’s and |Ψ^⊥^⟩. We then repeat the derivation of the Lehmann lower bound expression starting from the eigenvalue eq 2.28, replacing the full Hamiltonian *Ĥ* with the Hamiltonian of eq 3.1. By definition
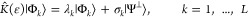
3.7and
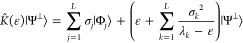
3.8

The Lehmann eq 2.28 remains as before. Multiplying it by the bra ⟨Φ_*k*_| and rearranging gives
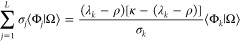
3.9

We then use the notation
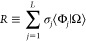
3.10so that
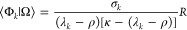
3.11

Inserting this into left-hand side of eq 3.9 leads to the eigenvalue equation
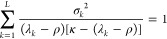
3.12

Comparing with eq 3.4 we identify κ + ρ with ε and ρ with *x*. This Lehmann equation is exact for the Hamiltonian *K̂*(ε) and is valid also for Coulombic systems. In other words, the PM method is identical to the Lehmann method for systems with Lanczos basis functions as may be inferred by comparing the PM equation and the Lehmann eigenvalue equation for Lanczos systems as in eq 2.37. If however one uses the Lehmann eq 2.28 for systems that cannot use the Lanczos construct such as Coulombic systems, then the PM method gives superior results as shall be exemplified below. However, the PM method is identical to Lehmann’s equation provided that one uses it with the matrix Hamiltonian rather than the full Hamiltonian.

This then complements the previous proof of how one obtains lower bounds. Let us suppose that, as before, L* is the highest state for which the interleaving property of the Ritz and exact eigenvalues holds. We then use ε_L*_^–^ as the Lehmann pole. The lower bound property of the Lehmann equation remains valid so that we know that
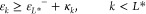
3.13

Finally, before closing this subsection, let us mention two further results that are instrumental to the next one. First, we notice that in the enlarged space, the eigenvector of the auxiliary Hamiltonian *K̂*(ε) with eigenvalue ε takes the simple form
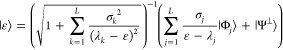
3.14as can be readily verified by inspection. Second, with some straightforward algebra, it is possible to recast the solutions for the eigenvalues other than ε as
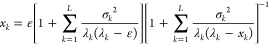
3.15and to write the associated eigenvectors in a form
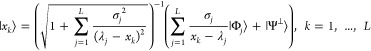
3.16that closely parallels eq 3.14. We will make use of these expressions in the next section where we shed light on the relationship between eq 3.4 and our recent findings.^14−16^

### 3.3 Improved Self-Consistent Lower Bound Theory Using the Hamiltonian of Equation 3.1

We may now also show that our previous improvements of Temple’s theory as described in refs^14,16^ and eq 2.26 are also identical to eq 3.4. Choosing the parameter ε to be the *k*-th eigenvalue of the Hamiltonian (*Ĥ*), the eigenfunction of the Hamiltonian *K̂*(ε) associated with this eigenvalue is denoted as |ε_*k*_⟩ and the eigenvectors associated with the remaining roots of eq 3.4 as |*x*_*j*_⟩ (see eqs 3.14 and 3.16, respectively). Similar to the development in Section 2 of this paper, we may rewrite each Ritz eigenvalue as
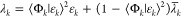
3.17where the residual energy of the Hamiltonian *K̂*(ε) becomes
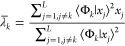
3.18

We then note that for the extended Hilbert space of the Hamiltonian *K̂*(ε), we have the identity
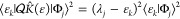
3.19but equivalently
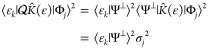
3.20so that with our construct
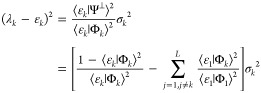
3.21

In view of eqs 3.19 and 3.20, we derive the analogue of the Lanczos relation of eq 2.25
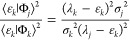
3.22and in view of eq 3.17
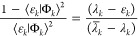
3.23

Putting this all into eq 3.21 and rearranging, we get the identity
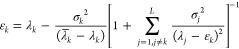
3.24and this has the same form as eq 2.26 without invoking a Lanczos basis set. To turn this into a practical expression, it is necessary to estimate the residual energy as defined in eq 3.18, which differs from the residual energy as defined in eq 2.15. For this purpose, one needs lower bounds to the roots *x*_*k*_ and this could follow the same procedure as above using the improved Weinstein lower bounds, which now may be also derived by assuming as in Weinstein theory that ⟨ε_*k*_|Φ_*k*_⟩^2^ ≥ 1/2 to find that
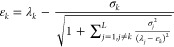
3.25

Alternatively, one could use the Bazley-related lower bounds to the corresponding eigenvalues of the Hamiltonian.

To summarize this section, we have two main results. The first and most important one is the practical one. Given the “Lehmann pole”, we obtain lower bounds to eigenvalues without the need to compute the full *Ĥ*^2^ matrix; all one needs are diagonal elements of it. Second, we have demonstrated the identity of the Lehmann lower bound expression with the self-consistent Temple lower bound expression and both are in principle exact. It remains to show that this methodology gives lower bounds for Coulombic systems that are superior to those obtained through the “standard” Lehmann methods. This is demonstrated in the next section.

## 4 Applications

### 4.1 He Atom Basis Set

To demonstrate the practicality of the theory, we consider first the ground state of the He atom. Our initial normalized function will be
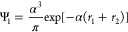
4.1with *r*_1_, *r*_2_ the distances of the electrons from the nucleus and the variational parameter α was chosen in all the computations as the value α = 27/16 which minimizes ⟨Ψ_1_|*Ĥ*|Ψ_1_⟩. The basis set was constructed using the scaled Schrödinger approach of Nakatsuji.^25^ The scaling function (with *r*_12_ the distance between the electrons) was chosen to be
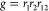
4.2as this was the easiest one to manipulate and compute using Maple. The Hamiltonian is
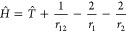
4.3and following Hylleraas,^27^ the kinetic energy operator is
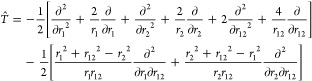
4.4

The volume integral is
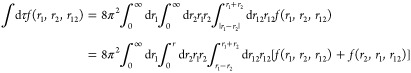
4.5

If as is often the case that
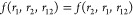
4.6so that the volume integral may be simplified to
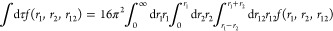
4.7

As already mentioned, the basis set is constructed using the scaled Schrödinger equation. Thus, the second normalized function will be
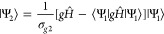
4.8with
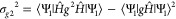
4.9

The third function will then be
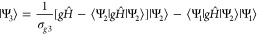
4.10with
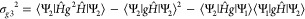
4.11and one continues in this fashion to build up the basis set. Note explicitly that although constructed similarly to the Lanczos algorithm, this procedure does not lead to a tridiagonal representation of the Hamiltonian.

In our computation, due to our use of Maple, we were limited to small dimensionality. Even for the seven-dimensional computation, the second lowest eigenvalue is higher than ε_3_ so that the lower bound computation is limited to the ground state. The same holds true of course for the Lehmann lower bound where even at *L* = 7, one finds only one negative eigenvalue which gives the lower bound to the ground-state energy.

### 4.2 Lower Bounds to the Ground-State Energy of He

**Figure 2:**
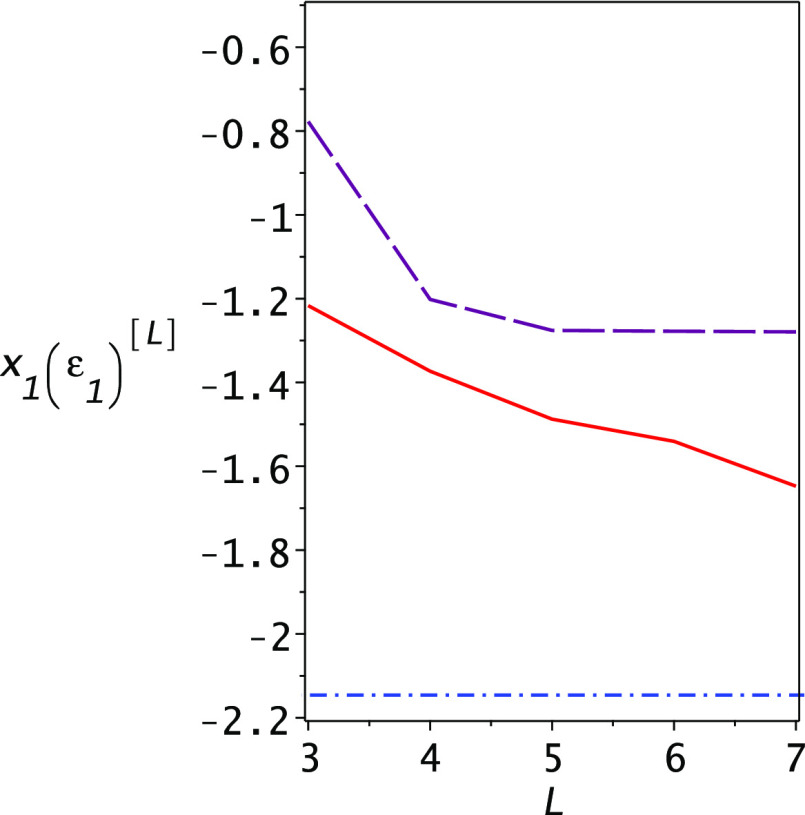
Dependence of the eigenvalue *x*_1_(ε_1_) on the dimensionality *L* of the basis set used for computation of the Helium atom energies (solid red line). Shown also is the second Ritz eigenvalue λ_2_ as a function of *L* (long dashed purple line); it is always larger than *x*_1_(ε_1_) and both slowly converge toward the energy ε_2_ (shown as the horizontal dashed–dotted blue line) from above. This demonstrates that one may use the energy ε_2_ as a lower bound to *x*_1_(ε_1_) and thus obtain lower bounds to the ground-state energy.

The lower-bound property is based on the observation that the solutions of eq 3.4 are monotonically decreasing functions of the dimensionality. This is shown for the specific case of the Helium atom with our chosen basis set in Figure 2 where we plot the dependence of *x*_1_(ε_1_) on the dimensionality of the basis set as it changes from 3 to 7. The monotonic decrease is the same when changing the argument in the vicinity of the ground-state energy. One does not need to know the exact ground-state energy to ascertain that the eigenvalue decreases with increasing dimensionality. As described in Section 3, it is the observation that the eigenvalue *x*_1_ is “trapped” between the Ritz eigenvalue λ_2_ and the exact eigenstate ε_2_, which allows us to replace *x*_1_ in eq 3.4 with ε_2_ or a lower bound to it such that the resulting lowest root of eq 3.4 will be a lower bound to the ground-state energy.

**Figure 3:**
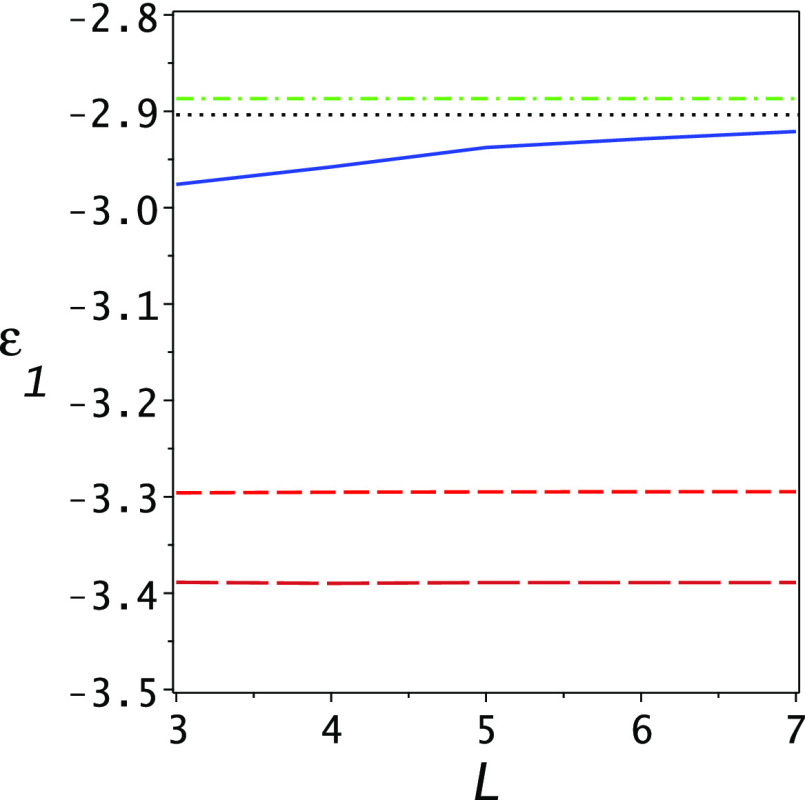
Lower bounds for the ground-state energy of the Helium atom as functions of the dimensionality of the basis set. The lowest (brown) long dashed line is the Temple lower bound, the dashed (orange) line is the Lehmann lower bound, and the solid blue line is the present PM lower bound. The black dotted line is the exact ground-state energy and the upper dashed–dotted (green) line is the Ritz eigenvalue for the ground state. Note the essential improvement of the lower bound obtained using the present PM theory.

**Figure 4:**
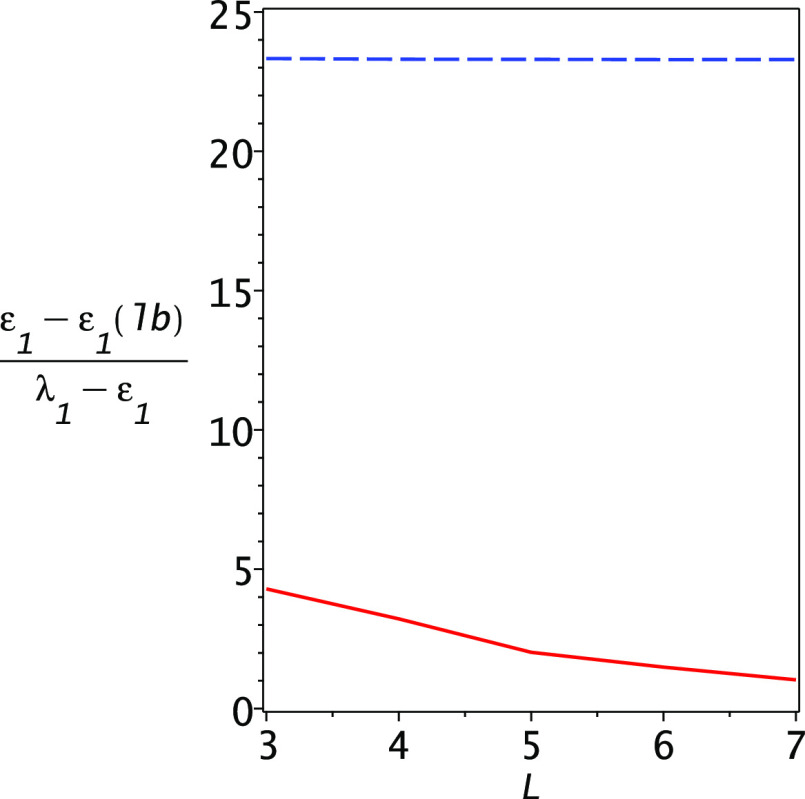
Gap ratios for the Lehmann and PM lower bounds for the Helium atom. Note that the PM lower bounds are roughly as accurate as the Ritz upper bounds, while the Lehmann lower bounds do not come close.

To test the new theory and compare it with Lehmann, we use the following strategy. For the “standard” Lehmann computation, we choose the best possible Lehmann pole: ε_2_ = −2.14597405. This is of course an idealization, typically if one knows one state, one knows the other and certainly if ε_2_ is known then so is ε_1_. In a “realistic” scenario, the Lehmann pole for the first excited state would be given by a Weinstein- or Bazley-type lower bound, but for the sake of understanding the new theory without adding in other sources of approximate values, we make this choice. Similarly, the value of ε_2_ was used to obtain the Temple lower bound as well as the PM lower bound derived from *x*_1_(ε_2_). The resulting lower bounds as well as the Ritz upper bounds are shown in Figure 3. One notes the essential improvement of the PM lower bound (solid blue line), which becomes competitive with the Ritz upper bound when the dimensionality reaches 7. This is further exemplified in Figure 4 where the gap ratio of the lower bound to the upper bound  is plotted as a function of dimensionality for the Lehmann (upper blue dashed line) and PM (lower solid red line) lower bounds. At *L* = 7, the PM gap ratio is 1.03.

One notes that in the range 3 ≤ *L* ≤ 7 the Ritz upper bound and the Temple and Lehmann lower bounds hardly change. The basis set we chose is not optimal in this sense, but critically, as the dimension is increased, the Ritz upper bounds to the excited states improve significantly, as may be seen for the first excited state in Figure 2. It is this improvement in the Ritz eigenvalues and variances of the excited states which leads to the significant improvement of the PM lower bound with dimensionality as shown in Figure 3.

### 4.3 H Atom

The energy levels of the hydrogen atom are known analytically, yet it serves as a good “playground” for studying lower bounds for this simplest of Coulombic systems. The Hamiltonian for the hydrogen atom is in atomic units (*r* is the electron proton distance)
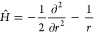
4.12The ground-state wavefunction is
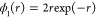
4.13and the ground-state energy is
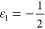
4.14

To test the lower bound expressions, we used the normalized antisymmetric harmonic oscillator basis set
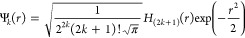
4.15such that
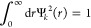
4.16and *H*_(*k*)_(*r*) is the *k*th order Hermite polynomial. This allows us to readily set up the Hamiltonian and Hamiltonian squared matrices and so test the various lower bound theories.

**Figure 5:**
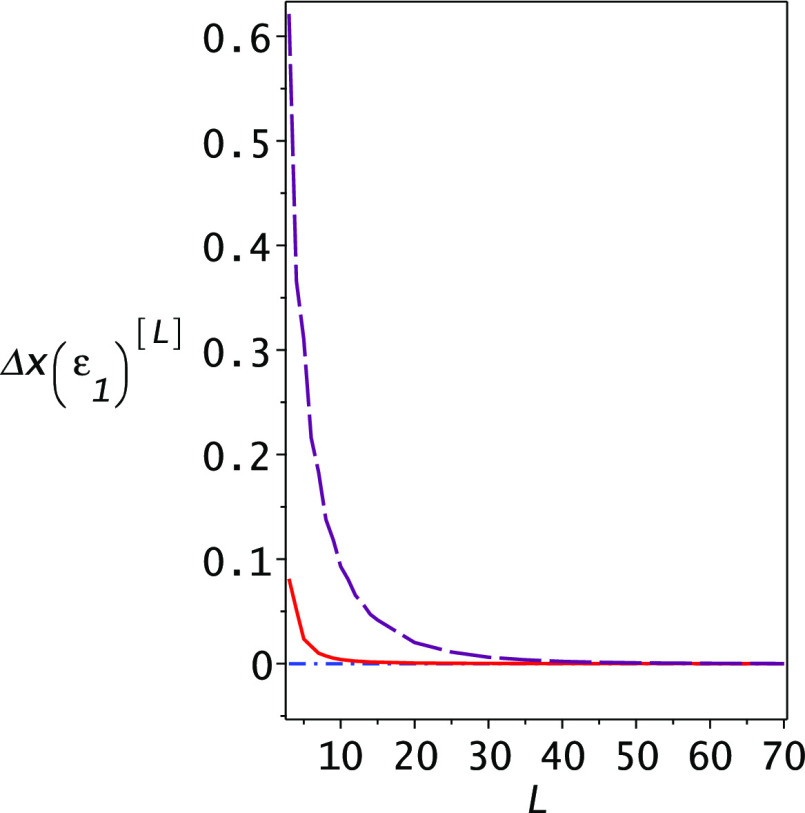
Dependence of the eigenvalue *x*_1_(ε_1_) on the dimensionality *L* of the computation for the Hydrogen atom using a Gauss Hermite basis set. The upper dashed (purple) line shows the difference λ_2_ – *x*_1_(ε_1_) between the Ritz upper bound for the second state and the eigenvalue *x*_1_(ε_1_) while the lower (red) solid line shows the difference *x*_1_(ε_1_) – ε_2_ between the eigenvalue *x*_1_(ε_1_) and the exact second-state energy ε_2_. Both lines are always positive, demonstrating that the second-state energy is indeed a lower bound to the eigenvalue *x*_1_(ε_1_).

As in the case of the Helium atom, we show in Figure 5 that *x*_1_(ε_1_) is a monotonically decreasing function of the dimensionality, ultimately going down to ε_2_. Here, we plot both the difference between the second Ritz eigenvalue and *x*_1_(ε_1_) (top, brown long dashed line) and the difference between *x*_1_(ε_1_) and the second excited-state energy (−1/8) (bottom red solid line) as functions of the dimensionality. The dotted–dashed blue line is 0, which should be the limit of both lines with increasing dimensionality. For all values, the pole *x*_1_(ε) is in between the Ritz eigenvalue λ_2_ and the exact energy ε_2_.

**Figure 6:**
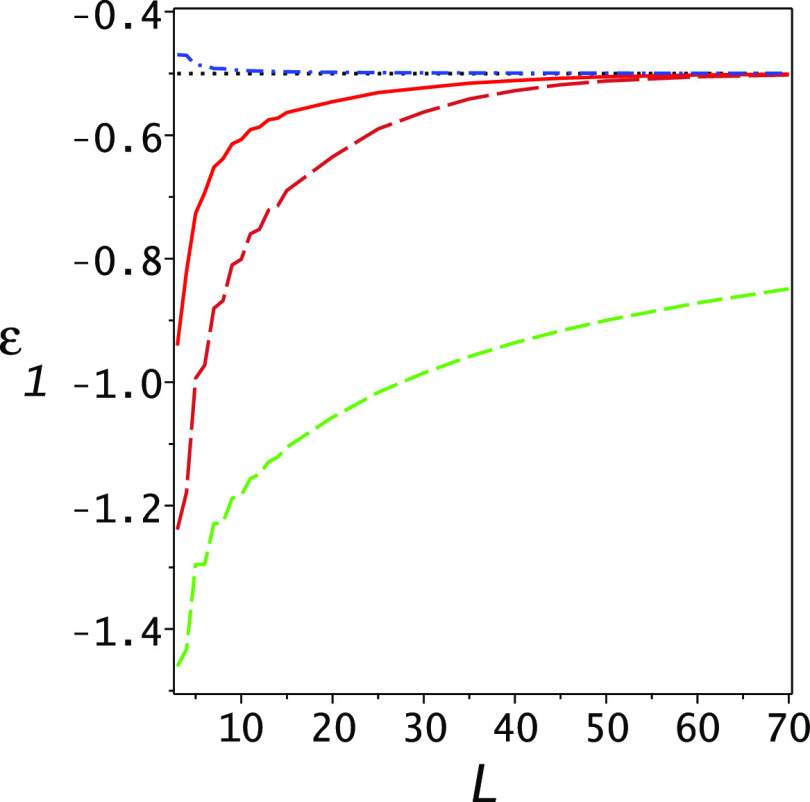
Lower bounds for the ground-state energy of the hydrogen atom as functions of the dimensionality of the basis set. The top dashed–dotted (blue) line is the Ritz upper bound, the (black) horizontal dotted line shows the exact ground-state eigenvalue, the solid (red) line is the present PM lower bound, the long dashed (brown) line is the Lehmann lower bound, and the dashed (green) line is the Temple lower bound. Note the superiority of the PM lower bound. The various bounds were computed for *L* = 3, 4, ..., 14, 15 and then for *L* = 20, 25, 30, 35, 40, 45, 50, 60, 70. The small oscillations at the lower dimensionality are a reflection of the basis set chosen. Adding in a new even function (*k* even in eq 4.15) improves the Ritz upper bounds more than adding another odd function.

**Figure 7:**
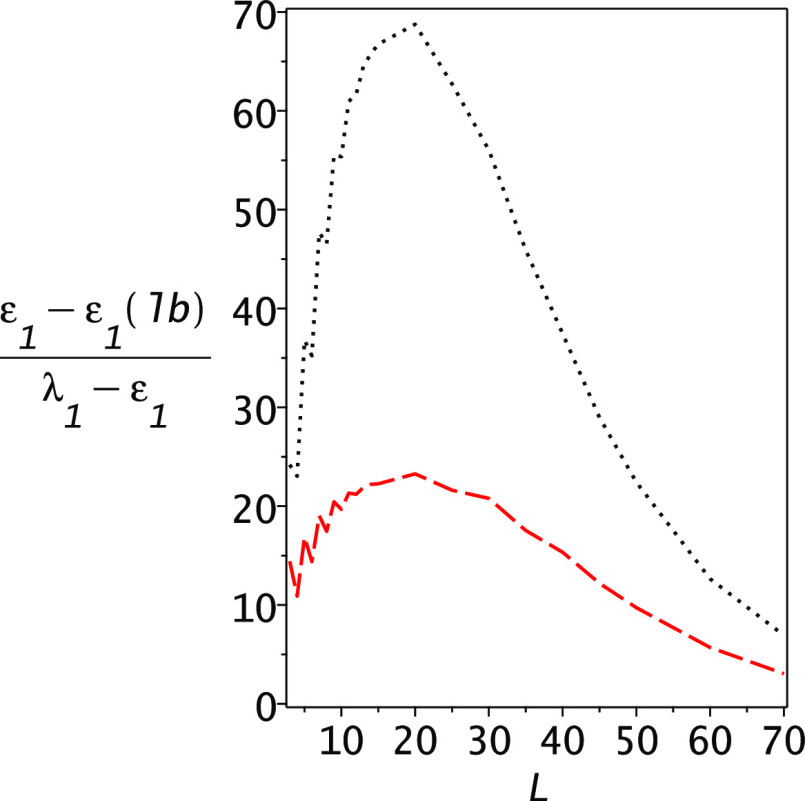
Gap ratios for the Lehmann and PM lower bounds for the hydrogen atom. The upper dotted line is for the Lehmann lower bound and the lower dashed line is the PM lower bound gap ratio. Note that the gap ratios of the PM lower bounds are substantially lower than those of the Lehmann lower bounds. At *L* = 70, the error in the Ritz upper bound is 0.00033, while the PM gap ratio is 3.05 and the Lehmann gap ratio is 6.93.

Then, we compute as a function of *L* the Temple, Lehmann, and PM lower bounds, in all of them using ε_2_ as the Lehmann pole energy. The results are shown in Figure 6 and one notices that PM theory is again superior to the other lower bounds. In Figure 7, we plot the gap ratios for the Lehmann and PM lower bounds; at its worst, the PM gap ratio is ca. 23 and one sees that it improves significantly with the increasing dimensionality of the basis set, reaching 3.05 when *L* = 70.

**Figure 8:**
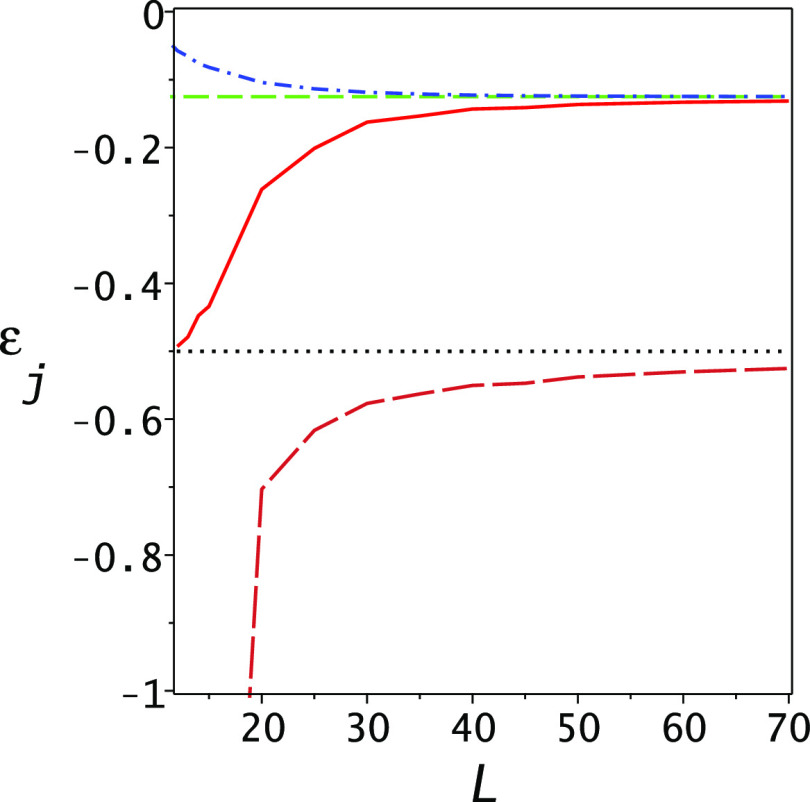
Lower bounds for the first excited state of the Hydrogen atom using ε_3_ = −1/18 as the Lehmann pole. The horizontal lines show the numerically exact first and second eigenvalues (−1/2, −1/8), the dashed lower (orange) line is the PM lower bound for the ground state, and the solid (red) line is the PM lower bound for the second state. The (blue) dashed–dotted line shows the second Ritz eigenvalue, which bounds the second state from above. As noted in the text, the lower bound shown here for the second state is superior to the same obtained from the “standard” Lehmann theory.

With our choice of basis set, for *L* ≥ 12 the second eigenvalue has the property that ε_2_ ≤ λ_2_ ≤ ε_3_ so that from *L* = 12, using ε_3_ as the “Lehmann pole”, one will get lower bounds for the ground and first excited state. This is shown in Figure 8 where the two horizontal lines are the ground and first excited-state energies while the lower dashed line is the PM lower bound to the ground state and the solid red line the PM lower bound to the second state. Comparing with Figure 6, one notes that the ground-state lower bound here is not as good as the one obtained with ε_2_ as the Lehmann pole. The reason is quite clear; the Ritz eigenvalue λ_2_ converges more rapidly than λ_3_ so that the lower bound to *x*_2_(ε_1_) given by ε_3_ is worse than the lower bound of ε_2_ and compared to *x*_1_(ε_2_). However, as may be seen from the plot, one is getting a rather “decent” lower bound for the second state −0.1314734 at *L* = 70 as compared to −0.125. Not as good as the Ritz upper bound (−0.124108), the gap ratio at *L* = 70 is 36.45, but the PM lower bound for the first excited state is much more accurate than the Lehmann lower bound, which is −0.166806 with a gap ratio of 235.4 under the same conditions.

## 5 Discussion

This work presents significant progress in lower bound theory

1. A simple polynomial equation has been derived for the Lehmann lower bound based on the use of a Lanczos basis set.
2. Lower bound theories were shown to have their origin in a generalized Cauchy–Schwartz inequality.
3. Using a finite Hamiltonian representation which is guaranteed to have one eigenvalue of the operator Hamiltonian, the polynomial equation derived for the Lehmann lower bound based on the Lanczos construct was generalized and shown to be valid even when the Lanczos structure cannot be constructed as in Coulombic systems.
4. The computational expense was significantly reduced since only Ritz eigenvalues and their associated standard deviations are needed to construct the lower bounds instead of the construction of the full matrix of *Ĥ*^2^ as in “standard” Lehmann theory.
5. The same derivation showed that both the Lehmann and the recent self-consistent lower bound method developed by the authors^14,15^ are within the present context of the finite Hamiltonian construct, identical.
6. The resulting PM theory was shown to be robust for the hydrogen and helium atoms and superior to any of the other lower bound theories. Lower bounds for Coulombic systems were demonstrated to have accuracy similar in quality to the Ritz upper bounds. The PM lower bound theory was implemented also for an excited state.

However, there remain difficult challenges ahead. Obtaining a Ritz upper bound is easy and straightforward. All that is needed is to construct the Hamiltonian matrix and diagonalize it. Deriving lower bounds is more complex. Even within the present simplified framework, one still needs to verify that the *x*_*k*_’s are monotonically decreasing functions of the dimensionality of the basis set used and it is necessary to compute the standard deviations associated with the Ritz eigenvalues. This implies the need to compute not only the Ritz eigenvalues but also their eigenfunctions. Perhaps, and this will be considered in future computations, it is not necessary to obtain the full orthogonal diagonalization matrix but only those of the first few dozen Ritz states to construct the matrix Hamiltonian and still obtain “good” lower bounds. But it will still be necessary to obtain the associated eigenfunctions of these lowest lying states, increasing the numerical cost of the computations.

However, the real challenge is not obtaining the eigenfunctions. A critical element in the theory is obtaining an accurate lower bound to the so-called “Lehmann pole”. In the present paper, we took the easy road, by using the known excited-state energies for the helium and hydrogen atoms. As stressed in the paper, the reason we did this was to provide a fair and unbiased comparison of the different lower bound theories. We saw that the present PM theory is superior to any other, yet the quality of the lower bound depends critically on the choice of the Lehmann pole. A “standard” methodology would be to use the Weinstein lower bound. For Hubbard-like Hamiltonians, we have shown^14^ that this choice is sufficient for obtaining tight lower bounds. The same is true for atoms as long as one is considering the ground state. However, the rapid reduction of the level spacing between excited states, as is the case in hydrogen, helium, and lithium,^11^ presents a serious challenge. Although we have shown how to improve upon the Weinstein lower bound, as for example in eq 2.27, the implementation is based on the assumption that the diagonal overlap matrix element squared is greater than 1/2. The challenge is to know when this assumption holds. In the “standard” Weinstein theory, one must consider the corresponding Ritz eigenvalue and know that it is the closest to the true eigenstate of the Hamiltonian under consideration. This is especially difficult when eigenstates come close together as for excited electronic states of atoms. Here, one must show that the Ritz eigenvalue is closest to the eigenvalue of the matrix Hamiltonian under consideration. These eigenvalues do not necessarily bunch together and this is an advantage; however, one does need to construct an objective criterion which would enable knowing that the overlap condition is valid, and this is not a trivial task.

Another challenge is understanding the choice of the basis set. Even the Ritz upper bound depends on the choice of the basis set. Different basis sets could give different Ritz upper bounds and PM lower bounds. For example, even in the present application of the theory to He, we made a specific choice of the exponent α in the initial wavefunction. In principle, for a given dimension of the basis set, one could vary α to minimize the Ritz eigenvalue for the ground state. One could also consider maximizing the PM lower bound for the ground state via variation of α. The two variations need not give the same value of the parameter. Different values imply different basis sets. The question of the “best” basis set remains open to both analytical as well as numerical research.

## Figures and Tables

**Figure 9:**
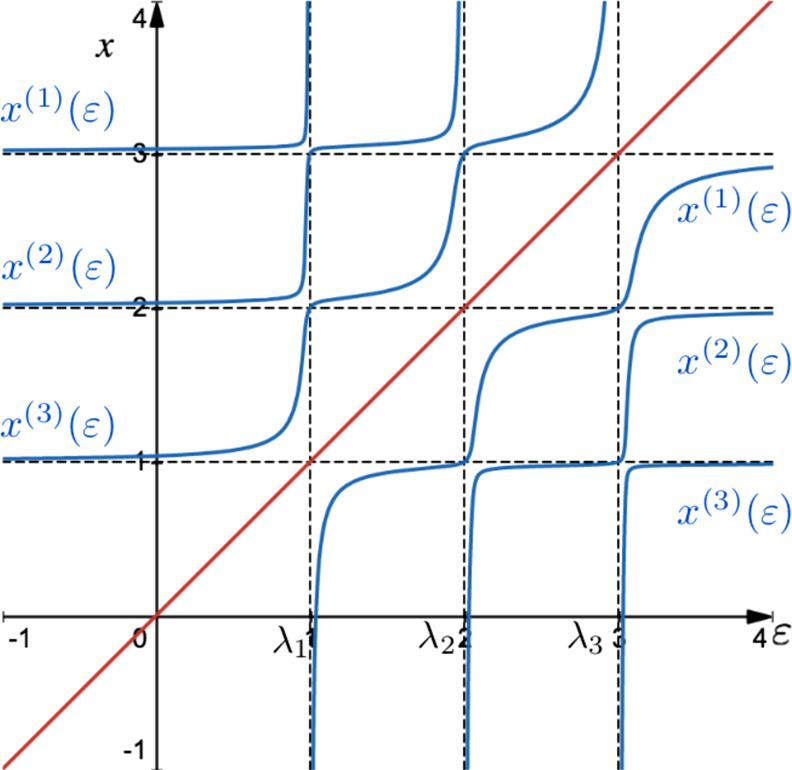
Eigenvalues of the auxiliary matrix  for a model three-state system with λ_*k*_ = 1, 2, 3 and σ_*k*_ = 0.20, 0.25, 0.30. The blue lines denote the continuous, monotonic functions *x*^(*k*)^(ε) labeled according to the position of their pole (ε = λ_*k*_) and the red line is the trivial eigenvalue ε.

**Figure 10:**
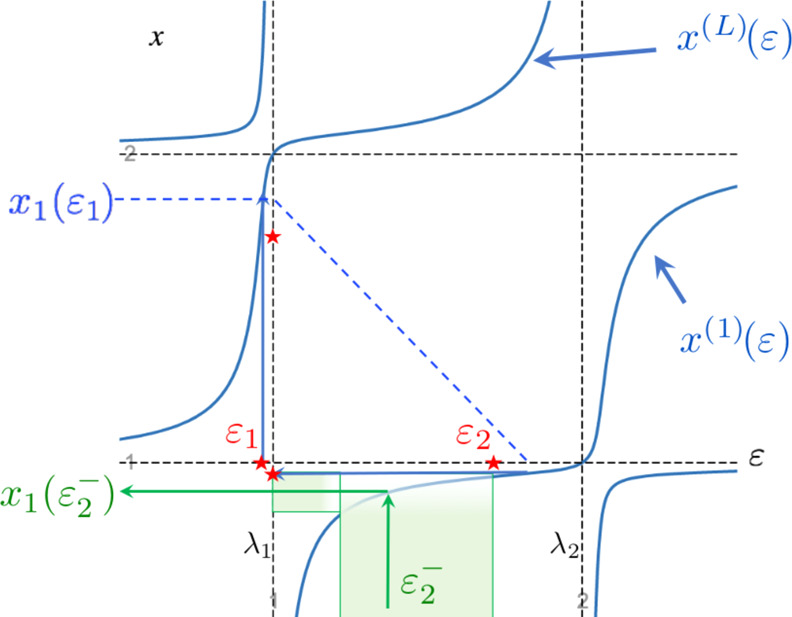
Close-up of Figure 9 showing in detail the region [λ_1_, λ_2_] relevant for calculating ground-state lower bounds according to the procedure described in the manuscript using a lower bound ε_2_^–^ to the first excited state. The red stars mark the exact eigenenergies on both the *x* and the *y* axes (colors and symbols as in Figure 1 of the main text).

## Appendix

### Monotonicity Properties of the Solutions of the PM Equation 3.4

We focus in this Appendix on the behavior of the eigenvalues of the auxiliary matrix  when ε approaches a Ritz eigenvalue λ_s_. We have already shown in the main text that ε is always an eigenvalue of the matrix; here, we show that when ε approaches λ_s_, the spectrum of the matrix  comprises the full set of Ritz eigenvalues plus a value tending to ±∞ for ε → λ_s_^∓^, respectively. The finite eigenvalues are smooth functions of ε that can be arranged to define solutions which are smooth and monotonically increasing on the whole real axis except for a pole at a Ritz eigenvalue. We use the pole position for their labeling, that is *x*^(*k*)^(ε), which is such a solution that is singular (only) for ε = λ_*k*_.

Let *q*_ε_(*x*) be the characteristic polynomial of the auxiliary matrix

where *p*_*L*_(*x*) = Π_*k*=1_^*L*^(λ_*k*_ – *x*) is the characteristic polynomial of the *L* × *L* upper left block of the matrix and *p*_*L*_^(*k*)^(*x*) = *p*_*L*_(*x*)/(λ_*k*_ – *x*) is a polynomial of degree *L* – 1. Henceforth, the Ritz eigenvalues are supposed to be non-degenerate; otherwise, additional care is required in the analysis below but the result remains essentially unchanged. Furthermore, as in the main text, we use the notation λ_*k*_ to denote the Ritz eigenvalues sorted in increasing order, λ_1_ < λ_2_ <···< λ_*L*_.

For *x* ≈ λ_*m*_, upon expanding *p*_*L*_(*x*) around *x* = λ_*m*_ and noticing that the required derivative is −*p*_*L*_^(*m*)^(λ_*m*_), one finds

where use has been made of *p*_*L*_^(*k*)^(λ_*m*_) = δ_*km*_*p*_*L*_^(*m*)^(λ_*m*_). Taking the limit of this expression for ε → λ_s_, we find

which shows that, for ε approaching λ_s_, the equation

defines an eigenvalue of the auxiliary matrix for any *m* = 1, ..., *L*. The remaining eigenvalue follows upon considering the limit for ε → λ_s_ and *x* → ±∞

which is seen to vanish provided

that is, *x* → ±∞ for ε approaching λ_s_ from below/above. Written in this way, *x* takes the form of a Temple–Lehmann bound in the worst scenario, that is, for the “pole” ε approaching the Ritz eigenvalue.

As for the finite solutions, it is evident from the above discussion that they are smooth around ε = λ_s_ with derivative ∂*x*/∂ε = σ_*m*_^2^/σ_s_^2^ > 0. Since they are always continuous when ε is not a Ritz eigenvalue and (as shown in the main text), they are also monotonic; they are in fact continuous functions that keep increasing when ε sweeps the real axis until they move beyond the highest eigenvalue, λ_*L*_. Suppose this happens for ε = λ_*k*–1_, then for ε approaching the next Ritz eigenvalue λ_*k*_, the solution tends to +∞ and, for ε > λ_*k*_, it increases again starting from −∞. We call this solution *x*^(*k*)^(ε) using the pole position for its labeling. Note that the divergence occurs for one solution at a time since the interlacing property guarantees that for ε not a Ritz eigenvalue, there exists one (and only one) eigenvalue in each of the intervals

(taking into account that ε is always an eigenvalue). Stated differently, we have the following theorem.

### Theorem

If the Ritz values λ_*k*_ are non-degenerate; the eigenvalues of the auxiliary matrix  other than ε can be arranged to define functions *x*^(*k*)^(ε) which are smooth and monotonically increasing on the whole real axis except for a pole at ε = λ_*k*_. Close to the latter, we have *x*^(*k*)^(ε) = *x*_*L*_(ε) for ε < λ_*k*_ and *x*^(*k*)^(ε) = *x*_1_(ε) for ε > λ_*k*_, and beyond that point *x*^(*k*)^ takes the next higher (lower) root every time ε crosses a Ritz eigenvalue to its right (left).

The functions defined in this way are monotonic in each connected domain, that is, separately for (−∞, λ_*k*_) and (λ_*k*_, +∞), but this is harmless for the method described in the main text. To see this, we first notice that, thanks to the symmetry between *x* and ε mentioned in the main text, the inverse of the function *x*^(*k*)^(ε) is some other function *x*^(*l*)^(ε). The specific value of *l* can be identified by inspecting the limiting value that *x*^(*k*)^ attains for ε → ±∞ since this gives the Ritz eigenvalue that makes the inverse singular (hence its label). We have *x*^(*k*)^(ε) → λ_*L*+1–*k*_^∓^ for ε → ±∞, hence *l* = *L* + 1 – *k*. In other words, for any , we have

The situation is illustrated in Figure 9 where we have plotted the solutions *x*^(*k*)^’s for a model three-state problem, along with the eigenvalue ε (red line), as functions of ε. The figure makes clear that discontinuity is a marginal problem and arises only when one insists on labeling the eigenvalues according to their magnitude: each function takes a different *x*_*k*_ in each interval (λ_*m*_, λ_*m*+1_), *m* = 1, 2,..., *L* – 1. Noteworthily, the symmetry between the *x*^(*k*)^’s and their inverse is apparent from the symmetry of the graph with respect to the main diagonal.

In the main text, in order to find a lower bound to the ground state, we have used *k* = *L*, hence the function *x*^(*L*)^ and its inverse *x*^(1)^, as illustrated schematically in Figure 10. Notice that on the left of λ_1_*x*^(*L*)^(ε) is the lowest lying root (apart from ε) and the same happens for *x*^(1)^(ε) when ε ∈ (λ_1_, λ_2_).

## References

[ref1] Reed M.; Simon B. Methods of Modern Mathematical Physics. Vol. IV: Analysis of Operators, 1st ed.; Academic Press: London, 1978.

[ref2] Temple G. The Theory of Rayleigh’s Principle as Applied to Continuous Systems Proc. Proc. R. Soc. London, Ser. A 1928, 119, 276 10.1098/rspa.1928.0098.

[ref3] Weinstein D. H. Modified Ritz Method. Proc. Natl. Acad. Sci. U.S.A. 1934, 20, 529–532. 10.1073/pnas.20.9.529. 16577632 PMC1076472

[ref4] Stevenson A. F. On the lower bounds of Weinstein and Romberg in quantum mechanics. Phys. Rev. 1938, 53, 199 10.1103/physrev.53.199.2.

[ref5] Lehmann N. J. Beiträge zur numerischen Lösung linearer Eigenwertprobleme. I. Z. Angew. Math. Mech. 1949, 29, 341–356.

[ref6] Lehmann N. J. Beiträge zur numerischen Lösung linearer Eigenwertprobleme. Z. Angew. Math. Mech. 1950, 30, 1–16. 10.1002/zamm.19500300101.

[ref7] Donchev A. G.; Kalachev S. A.; Kolesnikov N. N.; Tarasov V. I. The upper and lower bounds of energy for nuclear and Coulomb few-body systems. Phys. Part. Nucl. Lett. 2007, 4, 39–45. 10.1134/s1547477107010074.

[ref8] Nakashima H.; Nakatsuji T. How Accurately does the free complement wave function of a Helium atom satisfy the Schrödinger equation?. Phys. Rev. Lett. 2008, 101, 240406 10.1103/physrevlett.101.240406. 19113607

[ref9] Marmorimo M. G.; Almayouf A.; Krause T.; Le D. Optimization of the Temple lower bound. J. Math. Chem. 2012, 50, 833–842. 10.1007/s10910-011-9927-z.

[ref10] Marmorimo M. G. Comparison and union of the Temple and Bazley lower bounds. J. Math. Chem. 2013, 51, 2062–2073. 10.1007/s10910-013-0199-7.

[ref11] Lüchow A.; Kleindienst H. Accurate upper and lower bounds to the2Sstates of the lithium atom. Int. J. Quant. Chem. 1994, 51, 211–224. 10.1002/qua.560510405.

[ref12] Pollak E. An Improved Lower Bound to the Ground-State Energy. J. Chem. Theory Comput. 2019, 15, 1498–1502. 10.1021/acs.jctc.9b00128. 30753072

[ref13] Pollak E. A Tight Lower Bound to the Ground-State Energy. J. Chem. Theory Comput. 2019, 15, 4079–4087. 10.1021/acs.jctc.9b00344. 31244131

[ref14] Martinazzo R.; Pollak E. Lower bounds to eigenvalues of the Schrödinger equation by solution of a 90-y challenge. Proc. Natl. Acad. Sci. U.S.A 2020, 117, 16181–16186. 10.1073/pnas.2007093117. 32601240 PMC7368311

[ref15] Pollak E.; Martinazzo R. Self-consistent theory of lower bounds for eigenvalues. J. Chem. Phys. 2020, 152, 244110 10.1063/5.0009436. 32610989

[ref16] Ronto M.; Pollak E. Upper and lower bounds for tunneling splittings in a symmetric double-well potential. RSC Adv. 2020, 10, 34681–34689. 10.1039/d0ra07292c. PMC9056815 35514393

[ref17] Lanczos C. An iteration method for the solution of the eigenvalue problem of linear differential and integral operators. J. Res. Natl. Bur. Stand. 1950, 45, 255 10.6028/jres.045.026.

[ref18] Krylov A. N. On the numerical solution of equation by which are determined in technical problems the frequencies of small vibration of material systems. News Acad. Sci. USSR 1931, 7, 491–539.

[ref19] Nakatsuji H. Discovery of a General Method of Solving the Schrödinger and Dirac Equations That Opens a Way to Accurately Predictive Quantum Chemistry. Acc. Chem. Res. 2012, 45, 1480–1490. 10.1021/ar200340j. 22686372

[ref20] Beattie C. Harmonic Ritz and Lehmann bounds. Electron. Trans. Numer. Anal. 1998, 7, 18–39.

[ref21] Bazley N. W. Lower bounds for eigenvalues with application to the Helium atom. Phys. Rev. 1960, 120, 144–149. 10.1103/physrev.120.144. PMC222648 16590455

[ref22] Bazley N. W.; Fox D. W. Lower Bounds for Eigenvalues of Schrödinger’s Equation. Phys. Rev. 1961, 124, 483–492. 10.1103/physrev.124.483.

[ref23] Miller W. H. Improved Equation for Lower Bounds to Eigenvalues; Bounds for the Second-Order Perturbation Energy. J. Chem. Phys. 1969, 50, 2758–2762. 10.1063/1.1671442.

[ref24] Marmorino M. G. Eigenvalue lower bounds with Bazley’s special choice of an infinite-dimensional subspace. J. Math. Chem. 2011, 49, 1535–1543. 10.1007/s10910-011-9839-y.

[ref25] Nakatsuji H. Scaled Schrödinger equation and the exact wave function. Phys. Rev. Lett. 2004, 93, 030403 10.1103/physrevlett.93.030403. 15323808

[ref26] Fisk S. A very short proof of Cauchy’s interlace theorem for eigenvalues of Hermitian matrices. Amer. Math. Monthly 2005, 2, 118.

[ref27] Hylleraas E. A. The Schrödinger Two-Electron Atomic Problem. Adv. Quant. Chem. 1964, 1, 1–33. 10.1016/s0065-3276(08)60373-1.

